# Supplementary material for: Identification of photoperiod-induced specific miRNAs in the adrenal glands of Sunite sheep (Ovis aries)
Source: Front Vet Sci. 2022 Jul 22;9:888207. doi: 10.3389/fvets.2022.888207 (PMC9354845; doi:10.3389/fvets.2022.888207)
Supplement: Supplementary file 1 [file Data_Sheet_1.ZIP › Supplementary file/miRDeep2 file.zip!/miRDeep2 file/Novel_Structure/SP42A3/Novel_604.pdf]

Provisional ID	:	NC_019464.2_13068
Score total	:	10.4
Score for star read(s)	:	3.9
Score for read counts	:	2.7
Score for mfe	:	2.2
Score for randfold	:	1.6
Score for cons. seed	:	
Total read count	:	17
Mature read count	:	15
Loop read count	:	0
Star read count	:	2

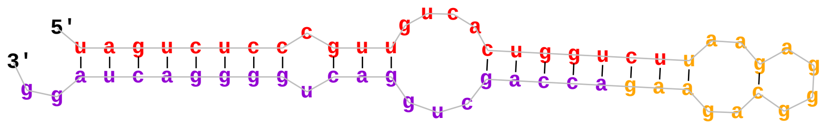

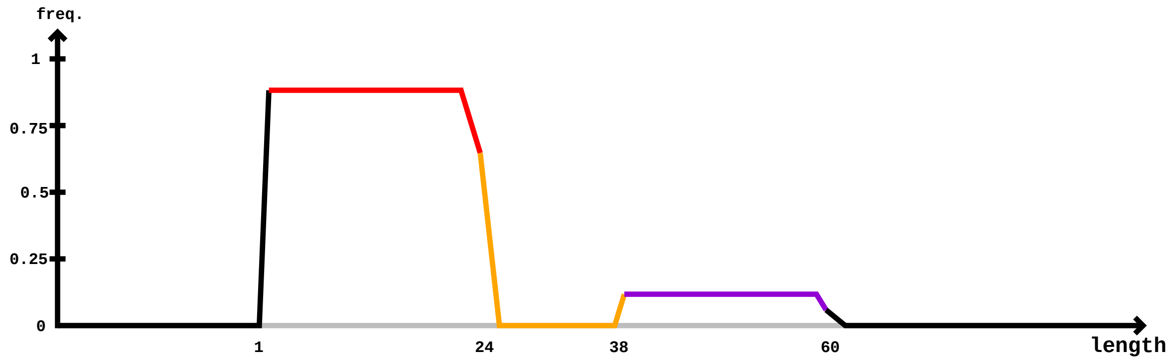

Mature **Star**

```
5'- aucccaccugggacaucuccuagucucccguugucacuggucuuaagagggcagaagaccagcuggacuggggacuaggagauaaccucccagaagugcuccuggacaacc   -3'    obs
    aucccaccugggacaucuccuagucucccguugucacuggucuuaagagggcagaagaccagcuggacuggggacuaggagauaaccucccagaagugcuccuggacaacc          exp
    .((((((((((((.((((((((((((((.(((....((((((((..(....)..))))))))...))).))))))))))))))....))))))..))).....))).....    reads    mm    sample
    ....................uagucucccguugucacuggu......................................................................    2        0     seq
    ....................uagucucccguugucacugguc.....................................................................    2        0     seq
    ....................uagucucccguugucacuggucu....................................................................    8        0     seq
    ....................uagucucccguugucacuggucuu...................................................................    3        0     seq
    .........................................................accagcuggacuggggacuag.................................    1        0     seq
    .........................................................accagcuggacuggggacuagg................................    1        0     seq
```
